# The use of race terms in epigenetics research: considerations moving forward

**DOI:** 10.3389/fgene.2024.1348855

**Published:** 2024-01-31

**Authors:** Dillon E. King, Pooja D. Lalwani, Gilberto Padilla Mercado, Emma L. Dolan, Johnna M. Frierson, Joel N. Meyer, Susan K. Murphy

**Affiliations:** ^1^ Nicholas School of the Environment, Duke University, Durham, NC, United States; ^2^ Department of Obstetrics and Gynecology, Duke University School of Medicine, Durham, NC, United States; ^3^ Department of Molecular Genetics and Microbiology, Duke University School of Medicine, Durham, NC, United States; ^4^ Department of Pharmacology and Cancer Biology, Duke University School of Medicine, Durham, NC, United States; ^5^ IDEALS Office, Duke University School of Medicine, Durham, NC, United States

**Keywords:** race, epigenetic mechanisms, health inequities, structural determinants of health, data transparency

## Abstract

The field of environmental epigenetics is uniquely suited to investigate biologic mechanisms that have the potential to link stressors to health disparities. However, it is common practice in basic epigenetic research to treat race as a covariable in large data analyses in a way that can perpetuate harmful biases without providing any biologic insight. In this article, we i) propose that epigenetic researchers open a dialogue about how and why race is employed in study designs and think critically about how this might perpetuate harmful biases; ii) call for interdisciplinary conversation and collaboration between epigeneticists and social scientists to promote the collection of more detailed social metrics, particularly institutional and structural metrics such as levels of discrimination that could improve our understanding of individual health outcomes; iii) encourage the development of standards and practices that promote full transparency about data collection methods, particularly with regard to race; and iv) encourage the field of epigenetics to continue to investigate how social structures contribute to biological health disparities, with a particular focus on the influence that structural racism may have in driving these health disparities.

## Introduction

One of the goals of researchers focused on human health in response to environmental stressors is to equitably promote excellent health for all. The epigenome is an interface between the genome and our environment, and as such is a prime target for exposures that can impact our health. The growing field of social epigenetics has demonstrated that adverse social environments and exposures can be associated with alterations in the epigenome (Cunliffe, 2016; Burris et al., 2016/01; Shields, 2017; Tung and Gilad, 2013; Evans et al., 2021). Given this, the epigenome is a mechanism that could be key in linking systemic racism to health disparities (Non, 2021; Martin et al., 2022). In human epigenetic studies, there are two common ways that race has been used in data analysis: as a covariate, or as a variable of interest. When race is assessed as a variable of interest, it is generally for the purpose of identifying health disparities. It is imperative that researchers keep assessing data with regard to race and other social factors in order to strengthen our understanding of social epigenetics and links between the epigenome and health disparities. In epigenetic research that is not focused on health disparities *per se*, such as the study of environmental exposures on epigenetics or associations of epigenetic patterning with disease outcomes, it is common practice to treat race as a covariate when looking for differences in epigenetic marks like DNA methylation or histone modifications. In this article, we call for the careful re-evaluation of the practice of using race as a covariate in epigenetic and epigenomic studies.

Despite the frequent use of the term “race” in biological and genomic research, it is more accurately defined as a socio-political rather than a biological classification (Yudell et al., 2016). The concept of biological races would indicate that humans are biologically distinct from one another but share a common ancestor. Humans do not have biological races, as human biological diversity does not align with racial populations (Graves, 2023). Modern use of the term “race” relies on social definitions that vary across cultures and over time rather than the identification of distinct biological groups (Roberts, 2011; Fuentes et al., 2019). In this paper, we exclusively discuss socially defined race. Terms frequently used in a biologic context such as *ancestry* or *ethnicity* reflect one’s relationship to other individuals in the context of genealogical history (Yudell et al., 2016). In contrast, *race* is a social concept that connects someone to a larger constructed group, often based on having physical, geographical, or social characteristics similar to others in that group (Yudell et al., 2016).

Given that racial classification systems have been developed from our socially constructed systems of stratification, power, and ideology, it is unlikely that an individual’s socially defined race *per se* influences epigenetic mechanisms (Smedley and Smedley, 2005; Yudell et al., 2016; Baker et al., 2017). This is because someone’s racial classification does not accurately represent the experiences of each and every individual within that racial grouping. However, the association of an individual’s socially defined race with many social determinants of health may likely result in broad differences in epigenetic markers across races. In this scenario, the category of *race* as a construct cannot be the ultimate driving factor influencing the epigenome, but is potentially associated with the causative factors such as physical or social aspects of their environment. For any such superficial associations between socially defined racial categories and differences in epigenetic marks (Xia et al., 2014/08; King et al., 2015; Song et al., 2015/12), it is imperative that the field focuses efforts towards a deeper understanding of the actual underlying causal mechanisms that lead race to be associated with health disparities–or at least explicitly discuss the possibilities. The field of epigenetics must seek to understand how social and environmental exposures combine with biology to affect the social distribution of disease (Williams and Sternthal, 2010).

In many epigenetic studies, socially defined race is used as a proxy for socioeconomic and sociodemographic variables, including financial stability, healthcare access and social stress, among others. However, individuals are not bound to experience certain stress levels or financial status based on their race alone, making race a poor proxy for many social metrics. This practice of substituting race for other social metrics may confound and distort data analyses. Using race as a proxy for socioeconomic status (SES) rather than collecting data on SES not only fails to accurately capture the relevant social factors intended for study, but can also reinforce negative racial stereotypes (Williams and Sternthal, 2010).

Socially defined race is also often used in epigenetic and epigenomic research as a proxy for ethnicity or ancestry. Given the large amount of genomic diversity within socially defined races, using the racial category with which an individual identifies will not accurately align with their genetics (Lewis et al., 2022). For example, Black people in the USA who are descended from enslaved individuals have varying degrees of European ancestry across regions within the USA: >30% European ancestry in West Virginia compared to less than 16% in South Carolina (Bryc et al., 2015). This is further counfounded by incorporating first generation Africans from all over the continent of Africa, who live in the USA and are considered by the USA census as *Black or African American*, though their ancestry will vary greatly from those who are descendents of enslaved individuals. Additionally, researchers must be mindful that socially defined racial categories differ between cultures when attempting to conduct research involving individuals from different regions of the world. For instance, between the USA and the UK, socially defined racial categories may carry different meanings and members of certain socially defined races may have entirely different genealogical history and ancestry in the USA compared to the United Kingdom.

Analyses that include genomic sequencing to categorize individuals into ethnic or ancestral groupings are also faced with the limitations of discretizing a continuous and very complex variable (Lewis et al., 2022). Ways in which ancestry data has been used in research shows that it is largely ambiguous and varies across studies, indicating that the field lacks consistent use and definitions of ancestry terms and interpretations (Dauda et al., 2023). The field of epigenetics should continue to conduct empirical studies on the role of genetics in epigenomics. Current efforts that conduct genomic sequencing in order to perform principle component analysis (PCA) for statistical corrections are more useful in that they do not rely solely on reported race. However, the use of PCA plots to determine genetically similar groupings based on statistics alone should recognize that the social experiences of individuals in these groups may not align and may conflate the data analyses. Additionally, using sequencing technologies to assign people to a single ancestral grouping and then inferring the influence this may have on epigenetics currently has inherent limitations given that researchers still do not have a good grasp on how individual and cumulative genetic differences influence epigenetic outcomes. Conflating socially defined race and ancestry through the practice of using race as a proxy for ancestry also has the potential to continue to perpetuate the false notion that race has a genetic basis.

Propagating the false narrative of race as a biologic or genetic construct rather than a social construct can perpetuate racist ideologies and exacerbate or create further disadvantages for members of specific socially defined races (Cerdeña et al., 2020). For example, associating race with genetics in healthcare can directly harm patients. When medical training leaves physicians with the idea that substantive genetic differences exist between races, this facilitates the implicit and explicit rationalization and justification of treating patients differently based on their race. This ultimately causes harm to individuals as it can result in alarming outcomes such as the administration of less pain medications due to false beliefs of racially-linked differences in pain tolerance (Hoffman et al., 2016), fewer preventative screenings for bone density measurements in Black women, the ultilization of race-adjusted glomerular filtration rate to assess kidney function which results in underdiagnosis of kidney disease in Black patients (Ahmed et al., 2021; Uppal et al., 2022), and many more examples. Additionally, attributing racial health disparities based on the false premise of racially-based genetic differences conflates the cause and the effect of health disparities and detracts from examining the underlying problems driving these differences, such as racism and systemic inequality.

Biomedical researchers, in their role as educators of students, can directly influence the training of future medical doctors and any biases they hold. In academic settings, many researchers and medical professionals may be unaware that race is a social construct and not quantifiably related to genetic ancestry. Basic biological research influences policy makers, medical doctor training, and has the potential to perpetuate harmful biases to the general public. When race is treated as a biological phenomenon rather than a social construct, it risks further perpetuating the incorrect notion that race has a genetically-defined rather a socially-defined origin.

Here we contend that the field of epigenetics needs to scrutinize its data collection methodology to target the environmental along with the social factors that contribute to the establishment, maintenance, and alterations of the epigenome. We also call for environmental epigenetic and epigenomic research to better understand how environmental exposures and experiences of social forces that differ between socially defined races can cause epigenetic changes resulting in racial health disparities. Such research efforts would be fortified by collaboration with social scientists and thoughtful data collection. This is particularly important to the field of epigenetics as these mechanisms could be the key to understanding how racism elicits a very real biologic response that could be, in part, responsible for the establishment of health disparities (Snyder-Mackler et al., 2020; Martin et al., 2022).

## Shortcomings in the use of race in epigenetic research

Scientists often fail to use race accurately in biological and genomic research in two key ways. First, by neglecting to distinguish between self-identified racial categories and assigned or assumed racial categories. Second, by the haphazard use and reporting of racial/ethnic variables in genetic research, that is, reliance on race without clearly articulating exactly what race represents (Yudell et al., 2016; Dauda et al., 2023). These oversights risk not only perpetuating a misperception of race as genetically based, but, by misclassifying race, may also reduce the validity and reliability of the scientific research. It is important to be aware that an individual’s reported race may differ based on whether it was assumed by someone else collecting demographic data or if it was self-reported. Additionally, an individual’s answer might change if their race is not accurately reflected as an answer to a survey question. The increasing number of people identifying as mixed-race further complicates this type of data collection. Without concrete knowledge of the influence of genetics and social factors on the epigenome, the practice of classifying an individual’s socially defined race and using it as a covariate in statistical models is problematically simplistic.

While it is useful to assess epigenetic patterning in a way that allows identification of health disparities (e.g., running multivariate models stratified by socially defined race), it is also important to address how race is employed in study design. Oftentimes race is simply included as one of many covariates in a multivariate model. The following examples are worth considering in conceptualizing why socially defined race as a covariate might confound data. It was common practice in the past for doctors to record race for their patients rather than have patients self-report. Race was therefore documented based solely on skin color, within limited categories. Rather than skin color, what if hair color or eye color was instead used in these models (Neal, 2008)? Would there be any biological rationale for doing so? Consider using the racial category *Asian and Pacific Islander* as a proxy for genetic ancestry. Individuals in these two groups have very different ancestry yet are often grouped together. Race is either inadequately defined in these situations or not defined at all. From these poorly conceived categorizations, particularly consequential oversights include failing to acknowledge how outcomes would differ in the event of racial misidentification, missing information for individuals who are multi-racial, if race has incongruous definitions across different individuals, or if an individual’s race is simply not one of the input options in the study design. Where race is used as a proxy for levels of stress, it would be far better to intentionally collect and use actual stress data, given that not all members of the same socially defined race will experience stress in the same way or to the same degree. The practice of using race as a covariate fundamentally limits and may even confound data interpretation.

## Structural racism drives racial health disparities

Studying the implications of socially defined race for health disparities should include explicit acknowledgement of the causal pathways through which race is associated with negative health outcomes. Disproportionate health outcomes are driven by factors associated with structural racism, not race itself (Phelan et al., 2010/03; Bailey et al., 2017; Bailey et al., 2021). Structural racism involves the “systemic racial exclusion from power, resources, opportunities, and wellbeing that is embedded in societal institutions” (Brown and Homan, 2022). A good example comes from the field of birth outcomes, in which a great deal of research indicates that race itself is not the driving risk (Chantarat et al., 2022; Ross, 2014; McAfee, 2017; Collier et al., 2021). In other words, Black women are not more likely to experience pre-term birth and higher rates of infant mortality because they are Black, but due to chronic stressors like racism and poverty. Structural racism may operate through disproportionate exposure to risks, such as social stressors, environmental racism, or through unequal access to material and social resources, including social and economic capital, freedom, autonomy, power and prestige (Brown and Homan, 2022).

It is important to note that the goal of research on socio-political experiences and their effects on health is not to place blame on the communities that are experiencing health disparities, but rather to identify the institutional structures and institutional policies that can be altered to help alleviate these unjust health disparities. Throughout history, research has often focused on attempting to connect health disparities with individual-level underlying health conditions and behavioral risk factors rather than institutional-level policies and experiences (McClure et al., 2020). Focusing on behavioral risk factors can result in blame being placed on individuals and their personal behaviors, such as poor diet or stress levels, rather than the actual structural issues that are to blame (Roberts, 2011). Attention is then drawn away from how racialized communities are more likely to experience greater levels of workplace hazards, experience low wage work, and lack access to high quality healthcare due to institutional policies (McClure et al., 2020). When researchers use a hyper-individual approach to identifying risk factors, the social causes of disease may be obscured. This approach allows society to both ignore how policy and inequality create a system in which not everyone can thrive, masking systemic oppression as a root cause of health inequities, and can further perpetuate false notions of inherent differences between socially defined races (McClure et al., 2020). In other words, the focus should be less on race in patient classification and identifying causal mechanisms, but rather more on race in terms of discerning the ways in which structural racism produces and exacerbates health inequities (McAfee, 2017).

## Toward achieving adequate measures of important social metrics

Quantifying structural racism is an important component of research that seeks to elucidate how policy and institutions impact health. Previous studies have demonstrated how structural racism and other state-level structural inequalities such as sexism, individually and jointly, shape health outcomes (Homan et al., 2021). As quantification of discrimination at the structural level becomes more sophisticated, researchers will have access to state and regional level estimates of structural inequality, which will allow them to model the ways socially defined race, gender, class, ability, and other individual-level characteristics interact with institutions to affect health outcomes (Brown and Homan, 2022; Homan et al., 2021; Hardeman et al., 2022; Atkins, 2014; James, 2022).

Social scientists and physical scientists must work together toward improving our understanding of how structural inequalities affect health outcomes. Social scientists’ development of measurements of structural discrimination could improve epigeneticists’ estimations of individual health outcomes. Others have already called for a more explicit paradigm shift with regard to scientists’ use of racial categories, including requiring journals to explain the use of classificatory terminology in studying human genetic diversity (Yudell et al., 2016). Epigenetics and social science research could also benefit from replicating existing studies that have relied on race, with substitution of more accurate and meaningful variables associated with socially defined race. For example, if *stress* is what *race* is intended to capture, then including measures of perceived racism, socioeconomic status, allostatic load, birth zip code, measurements of neighborhood deprivation, some measure of wealth or financial safety net, social network, and primary language would serve to better capture and characterize levels of stress.

## Epigenetics offers tools for understanding health disparities

The field of epigenetics promises to reveal much about the relationship between social, political, or environmental factors and their influence on health disparities. Epigenetics research has the potential to improve our understanding of how chronic stress, nutritional status, and socio-political structures such as structural racism and environmental racism can influence the health of individuals and impact health across generations (Breton et al., 2021; Salas et al., 2021; Chan et al., 2023). Epigenetic research is already examining the influence of nutritional status on the epigenome (Ideraabdullah and Zeisel, 2018; Gomez-Verjan et al., 2020), the links between DNA methylation and higher incidence rates of chronic pain in African Americans (Aroke et al., 2019), and epigenetic alterations associated with racial trauma (McDade et al., 2017; Grossi, 2020). Health disparities in cardiovascular disease (Kuzawa and Sweet, 2009) and exposures to environmental contaminants (Majnik and Lane, 2014; House et al., 2019) are of major interest in the field and can help provide insight into how structural racism embedded in our society can have negative health outcomes mediated though epigenetic mechanisms.

## Recommendations

Given the rapidly progressing nature of the field of health disparities research, best practices for incorporating socially defined race into epigenetics research will likely change over time. However, the intention here is to prompt researchers to think more critically as they design, plan, and carry out experiments, and that when deciding where to allocate time and resources, funding agencies acknowledge the limitations and implications of oversimplifying the role of race. We and others (Krieger, 2020; Chan et al., 2023; Lewis et al., 2023; National Academies of Sciences E and Medicine, 2023) have put forth reccomendations for carrying out genetic and epigenetic research in the context of socially defined race and health disparities. Our recommendations include:

1. Researchers should engage in dialogues about the definitions of race, what socially defined race does and does not represent, and why socially defined race is included as a covariate in study design. Bringing this topic to the forefront of pedagogy will help young scientists continue to push this field and think critically about this issue. Dedicating sessions in scientific conferences to discuss, learn, and understand these issues will help formalize best practices in the field of epigenetics. The hope is that this will stimulate conversations about how socially defined race is used in the field of environmental epigenetics research and how race in the resulting research may be interpreted by lawmakers and clinicians as well as how this might influence biases held by decision makers and further perpetuate health disparities.
2. If researchers choose to use socially defined race as a covariate in models, then a justification should be provided in the context of the scientific question being asked. In the case of using data from a previously established cohort with limited demographic data, researchers should acknowledge the weaknessess associated with the data available to them and be fully transparent about the methods of data collection regarding social metrics. Depending on the demographic data available for each cohort, some metrics could be extrapolated from zipcode data, such as levels of neighborhood deprivation. However, we recommend this is performed such that the researchers recognize the imperfections in extrapolating this data and work closely with social scientists when doing do.
3. Where socially defined race is used as a proxy for variables like stress, other data likely to be associated with stress should be collected; for example, levels of perceived racism, regional levels of structural discrimination, socioeconomic status, or zip code (Williams and Sternthal, 2010; The Use of Racial, 2005) that may be the explanatory variable that drives the association between socially defined race and alteration of the epigenome. Collaboration with social scientists may give more insight into tools researchers can use to collect better measurements of structural racism. Where socially defined race is used as a proxy for ancestry, researchers should confront the inherent limitations of this oversimplificiation. When utilizing genetic ancestry in epigenetic studies, researchers should engage with ethical frameworks for incorporating ancestry data in their analyses (Lewis et al., 2022; Lewis et al., 2023). Embracing an interdisciplinary framework in study designs will allow for the incorporation of the social and cultural drivers of health disparities and provide a better understanding of how the epigenome is being modified.
4. New cohorts should be inclusive, representative of many backgrounds, and include as much demographic data as possible so that researchers can assess interactions of multiple social and environmental stressors with chemical exposures. However, inclusion of enough minority participants in new cohorts to achieve sufficient statistical power will require overcoming distrust between communities and researchers. Establishing trust can be difficult and will be best accomplished when researchers are actively engaging with community and addressing topics of concerns to them (Masuda et al., 2011; Han et al., 2021/; Mikesell et al., 2013; Cook, 2008; Wallerstein and Duran, 2010; Gilmore-Bykovskyi et al., 2022). It is important that scientists approach community engagement with the goal of not just communicating science to the public but working to understand their concerns and include them in the scientific process. Research topics should connect with the social, cultural, and political context in which they reside.
5. In designing new studies, self-reported race (Lorusso and Bacchini, 2015) and ethnicity data should still be collected so that data stratified across socially defined races can be used to help identify racial disparities. With regard to these data, it is most important that researchers are transparent about how race and ethnicity variables are obtained, particularly regarding whether race data was codified by others or if it was self-reported. Researchers should provide detailed information about the demographic data collection process.
6. More research on the topic of epigenetics and racism is needed to understand the mechanistic links through which institutional racism can exert direct impacts on health, perpetuating health disparities. Funds, time, and resources need to be dedicated to research focused on how institutionalized racism and environmental racism can influence epigenetics and potentially influence health for generations.

## Data Availability

The original contributions presented in the study are included in the article/Supplementary Material, further inquiries can be directed to the corresponding author.

## References

[B2] Ahmed S. Nutt C. T. Eneanya N. D. Reese P. P. Sivashanker K. Morse M. (2021). Examining the potential impact of race multiplier utilization in estimated glomerular filtration rate calculation on African-American care outcomes. J. Gen. Intern Med. 36 (2), 464–471. 10.1007/s11606-020-06280-5 33063202 PMC7878608

[B3] Aroke E. N. Joseph P. V. Roy A. Overstreet D. S. Tollefsbol T. O. Vance D. E. (2019). Could epigenetics help explain racial disparities in chronic pain? J. Pain Res. 12, 701–710. 10.2147/JPR.S191848 30863142 PMC6388771

[B4] Atkins R. (2014). Instruments measuring perceived racism/racial discrimination: review and critique of factor analytic techniques. Int. J. Health Serv. 44 (4), 711–734. 10.2190/HS.44.4.c 25626225 PMC4389587

[B5] Bailey Z. D. Feldman J. M. Bassett M. T. (2021). How structural racism works — racist policies as a root cause of U.S. racial health inequities. N. Engl. J. Med. 384 (8), 768–773. 10.1056/NEJMms2025396 33326717 PMC11393777

[B6] Bailey Z. D. Krieger N. Agénor M. Graves J. Linos N. Bassett M. T. (2017). Structural racism and health inequities in the USA: evidence and interventions. Lancet 389 (10077), 1453–1463. 10.1016/S0140-6736(17)30569-X 28402827

[B7] Baker J. L. Rotimi C. N. Shriner D. (2017). Human ancestry correlates with language and reveals that race is not an objective genomic classifier. Sci. Rep. 7 (1), 1572. 10.1038/s41598-017-01837-7 28484253 PMC5431528

[B8] Breton C. V. Landon R. Kahn L. G. Enlow M. B. Peterson A. K. Bastain T. (2021). Exploring the evidence for epigenetic regulation of environmental influences on child health across generations. Commun. Biol. 4 (1), 769. 10.1038/s42003-021-02316-6 34158610 PMC8219763

[B9] Brown T. H. Homan P. A. (2022). Frontiers in measuring structural racism and its health effects. Health Serv. Res. 57 (3), 443–447. 10.1111/1475-6773.13978 35468217 PMC9108047

[B10] Bryc K. Durand E. Y. Macpherson J. M. Reich D. Mountain J. L. (2015). The genetic ancestry of African Americans, Latinos, and European Americans across the United States. Am. J. Hum. Genet. 96 (1), 37–53. 10.1016/j.ajhg.2014.11.010 25529636 PMC4289685

[B11] Burris H. H. Baccarelli A. A. Wright R. O. Wright R. J. (2016/01/01 2016). Epigenetics: linking social and environmental exposures to preterm birth. Pediatr. Res. 79 (1), 136–140. 10.1038/pr.2015.191 PMC4740247 26460521

[B12] Cerdeña J. P. Plaisime M. V. Tsai J. (2020). From race-based to race-conscious medicine: how anti-racist uprisings call us to act. Lancet 396 (10257), 1125–1128. 10.1016/s0140-6736(20)32076-6 33038972 PMC7544456

[B13] Chan M. H.-m Merrill S. M. Konwar C. Kobor M. S. (2023). An integrative framework and recommendations for the study of DNA methylation in the context of race and ethnicity. Discov. Soc. Sci. Health 3 (1), 9. 10.1007/s44155-023-00039-z 37122633 PMC10118232

[B14] Chantarat T. Van Riper D. C. Hardeman R. R. (2022). Multidimensional structural racism predicts birth outcomes for Black and White Minnesotans. Health Serv. Res. 57 (3), 448–457. 10.1111/1475-6773.13976 35468220 PMC9108042

[B15] Collier A.-rY. Ledyard R. Montoya-Williams D. Qiu M. Dereix A. E. Farrokhi M. R. (2021). Racial and ethnic representation in epigenomic studies of preterm birth: a systematic review. Epigenomics 13 (21), 1735–1746. 10.2217/epi-2020-0007 33264049 PMC8579936

[B16] Cook W. K. (2008). Integrating research and action: a systematic review of community-based participatory research to address health disparities in environmental and occupational health in the USA. J. Epidemiol. Community Health 62 (8), 668–676. 10.1136/jech.2007.067645 18621950 PMC2772828

[B17] Cunliffe V. T. (2016). The epigenetic impacts of social stress: how does social adversity become biologically embedded? Epigenomics 8 (12), 1653–1669. 10.2217/epi-2016-0075 27869483 PMC5289034

[B18] Dauda B. Molina S. J. Allen D. S. Fuentes A. Ghosh N. Mauro M. (2023). Ancestry: how researchers use it and what they mean by it. Front. Genet. 14, 1044555. 10.3389/fgene.2023.1044555 36755575 PMC9900027

[B19] Evans L. Engelman M. Mikulas A. Malecki K. (2021). How are social determinants of health integrated into epigenetic research? A systematic review. Soc. Sci. Med. 273, 113738. 10.1016/j.socscimed.2021.113738 33610974 PMC8034414

[B20] Fuentes A. Ackermann R. R. Athreya S. Bolnick D. Lasisi T. Lee S. H. (2019). AAPA statement on race and racism. Am. J. Phys. Anthropol. 169 (3), 400–402. 10.1002/ajpa.23882 31199004

[B21] Gilmore-Bykovskyi A. Croff R. Glover C. M. Jackson J. D. Resendez J. Perez A. (2022). Traversing the aging research and health equity divide: toward intersectional frameworks of research justice and participation. Gerontologist 62 (5), 711–720. 10.1093/geront/gnab107 34324633 PMC9154232

[B22] Gomez-Verjan J. C. Barrera-Vázquez O. S. García-Velázquez L. Samper-Ternent R. Arroyo P. (2020). Epigenetic variations due to nutritional status in early-life and its later impact on aging and disease. Clin. Genet. 98 (4), 313–321. 10.1111/cge.13748 32246454

[B23] Graves J. L. Jr. (2023). Favored races in the struggle for life: racism and the speciation concept. Cold Spring Harb. Perspect. Biol. 15 (8), a041454. 10.1101/cshperspect.a041454 37463717 PMC10411861

[B24] Grossi É. (2020). New avenues in epigenetic research about race: online activism around reparations for slavery in the United States. Soc. Sci. Inf. 59 (1), 93–116. 10.1177/0539018419899336

[B25] Han H.-R. Xu A. Mendez K. J. W. Okoye S. Cudjoe J. Bahouth M. (2021). Exploring community engaged research experiences and preferences: a multi-level qualitative investigation. Res. Involv. Engagem. 7 (1), 19. 10.1186/s40900-021-00261-6 33785074 PMC8008581

[B26] Hardeman R. R. Homan P. A. Chantarat T. Davis B. A. Brown T. H. (2022). Improving the measurement of structural racism to achieve antiracist health policy. Health Aff. 41 (2), 179–186. 10.1377/hlthaff.2021.01489 PMC9680533 35130062

[B27] Hoffman K. M. Trawalter S. Axt J. R. Oliver M. N. (2016). Racial bias in pain assessment and treatment recommendations, and false beliefs about biological differences between blacks and whites. Proc. Natl. Acad. Sci. U. S. A. 113 (16), 4296–4301. 10.1073/pnas.1516047113 27044069 PMC4843483

[B28] Homan P. Brown T. H. King B. (2021). Structural intersectionality as a new direction for health disparities research. J. Health Soc. Behav. 62 (3), 350–370. 10.1177/00221465211032947 34355603 PMC8628816

[B29] House J. S. Hall J. Park S. S. Planchart A. Money E. Maguire R. L. (2019). Cadmium exposure and *MEG3* methylation differences between whites and african Americans in the NEST cohort. Environ. Epigenet 5 (3), dvz014. 10.1093/eep/dvz014 31528362 PMC6736358

[B30] Ideraabdullah F. Y. Zeisel S. H. (2018). Dietary modulation of the epigenome. Physiol. Rev. 98 (2), 667–695. 10.1152/physrev.00010.2017 29442595 PMC5966714

[B31] James D. (2022). An initial framework for the study of internalized racism and health: internalized racism as a racism-induced identity threat response. Soc. Personality Psychol. Compass 16, e12712. 10.1111/spc3.12712

[B32] King K. Murphy S. Hoyo C. (2015). Epigenetic regulation of newborns’ imprinted genes related to gestational growth: patterning by parental race/ethnicity and maternal socioeconomic status. J. Epidemiol. Community Health 69 (7), 639–647. 10.1136/jech-2014-204781 25678712 PMC4466032

[B33] Krieger N. (2020). Measures of racism, sexism, heterosexism, and gender binarism for health equity research: from structural injustice to embodied harm—an ecosocial analysis. Annu. Rev. Public Health 41 (1), 37–62. 10.1146/annurev-publhealth-040119-094017 31765272

[B34] Kuzawa C. W. Sweet E. (2009). Epigenetics and the embodiment of race: developmental origins of US racial disparities in cardiovascular health. Am. J. Hum. Biol. 21 (1), 2–15. 10.1002/ajhb.20822 18925573

[B35] Lewis A. C. F. Molina S. J. Appelbaum P. S. Dauda B. Di Rienzo A. Fuentes A. (2022). Getting genetic ancestry right for science and society. Science 376 (6590), 250–252. 10.1126/science.abm7530 35420968 PMC10135340

[B36] Lewis A. C. F. Molina S. J. Appelbaum P. S. Dauda B. Fuentes A. Fullerton S. M. (2023). An ethical framework for research using genetic ancestry. Perspect. Biol. Med. 66 (2), 225–248. 10.1353/pbm.2023.0021 37755714

[B37] Lorusso L. Bacchini F. (2015). A reconsideration of the role of self-identified races in epidemiology and biomedical research. Stud. Hist. Philos. Biol. Biomed. Sci. 52, 56–64. 10.1016/j.shpsc.2015.02.004 25791919

[B38] Majnik A. V. Lane R. H. (2014). Epigenetics: where environment, society and genetics meet. Epigenomics 6 (1), 1–4. 10.2217/epi.13.83 24579939

[B39] Martin C. L. Ghastine L. Lodge E. K. Dhingra R. Ward-Caviness C. K. (2022). Understanding health inequalities through the lens of social epigenetics. Annu. Rev. Public Health 43 (1), 235–254. 10.1146/annurev-publhealth-052020-105613 35380065 PMC9584166

[B40] Masuda J. R. Creighton G. Nixon S. Frankish J. (2011). Building capacity for community-based participatory research for health disparities in Canada: the case of “partnerships in community health research”. Health Promot. Pract. 12 (2), 280–292. 10.1177/1524839909355520 21057046

[B41] McAfee S. (2017). Race is a social construct. Available at: https://centerforhealthprogress.org/blog/race-social-construct/.

[B42] McClure E. S. Vasudevan P. Bailey Z. Patel S. Robinson W. R. (2020). Racial capitalism within public health-how occupational settings drive COVID-19 disparities. Am. J. Epidemiol. 189 (11), 1244–1253. 10.1093/aje/kwaa126 32619007 PMC7337680

[B43] McDade T. W. Ryan C. Jones M. J. MacIsaac J. L. Morin A. M. Meyer J. M. (2017). Social and physical environments early in development predict DNA methylation of inflammatory genes in young adulthood. Proc. Natl. Acad. Sci. 114 (29), 7611–7616. 10.1073/pnas.1620661114 28673994 PMC5530653

[B44] Mikesell L. Bromley E. Khodyakov D. (2013). Ethical community-engaged research: a literature review. Am. J. Public Health 103 (12), e7–e14. 10.2105/ajph.2013.301605 PMC3828990 24134352

[B45] National Academies of Sciences E, Medicine (2023). Using population descriptors in genetics and genomics research: a new framework for an evolving field. United States: The National Academies Press, 240. 36989389

[B46] Neal K. C. (2008). Use and misuse of ‘race’ in biomedical research. J. Health Ethics 5 (1). 10.18785/ojhe.0501.08

[B47] Non A. L. (2021). Social epigenomics: are we at an impasse? Epigenomics 13 (21), 1747–1759. 10.2217/epi-2020-0136 33749316 PMC8819598

[B48] Phelan J. C. Link B. G. Tehranifar P. (2010/03/01 2010). Social conditions as fundamental causes of health inequalities: theory, evidence, and policy implications. J. Health Soc. Behav. 51 (1_Suppl. l), S28–S40. 10.1177/0022146510383498 20943581

[B49] Roberts D. E. (2011). Fatal invention: how science, politics, and big business re-create race in the twenty-first century. New York: New Press.

[B50] Ross J. (2014). Epigenetics: the controversial science behind racial and ethnic health disparities. Washington, D.C.: The Atlantic.

[B51] Salas L. A. Peres L. C. Thayer Z. M. Smith R. W. Guo Y. Chung W. (2021). A transdisciplinary approach to understand the epigenetic basis of race/ethnicity health disparities. Epigenomics. 13 (21), 1761–1770. 10.2217/epi-2020-0080 33719520 PMC8579937

[B52] Shields A. E. (2017). Epigenetic signals of how social disadvantage "gets under the skin": a challenge to the public health community. Epigenomics. 9 (3), 223–229. 10.2217/epi-2017-0013 28234017

[B53] Smedley A. Smedley B. D. (2005). Race as biology is fiction, racism as a social problem is real: anthropological and historical perspectives on the social construction of race. Am. Psychol. 60 (1), 16–26. 10.1037/0003-066x.60.1.16 15641918

[B54] Snyder-Mackler N. Burger J. R. Gaydosh L. Belsky D. W. Noppert G. A. Campos F. A. (2020). Social determinants of health and survival in humans and other animals. Science 368 (6493), eaax9553. 10.1126/science.aax9553 32439765 PMC7398600

[B55] Song M.-A. Brasky T. M. Marian C. Weng D. Y. Taslim C. Dumitrescu R. G. (2015/12/02 2015). Racial differences in genome-wide methylation profiling and gene expression in breast tissues from healthy women. Epigenetics 10 (12), 1177–1187. 10.1080/15592294.2015.1121362 PMC4844220 26680018

[B1] The use of racial, ethnic, and ancestral categories in human genetics research. Am. J. Hum. Genet. (2005). 77 (4), 519–532. 10.1086/491747 16175499 PMC1275602

[B56] Tung J. Gilad Y. (2013). Social environmental effects on gene regulation. Cell Mol. Life Sci. 70 (22), 4323–4339. 10.1007/s00018-013-1357-6 23685902 PMC3809334

[B57] Uppal P. Golden B. L. Panicker A. Khan O. A. Burday M. J. (2022). The case against race-based GFR. Dela J. Public Health 8 (3), 86–89. 10.32481/djph.2022.08.014 PMC9495470 36177174

[B58] Wallerstein N. Duran B. (2010). Community-based participatory research contributions to intervention research: the intersection of science and practice to improve health equity. Am. J. Public Health 100 (1), S40–S46. 10.2105/ajph.2009.184036 20147663 PMC2837458

[B59] Williams D. R. Sternthal M. (2010). Understanding racial-ethnic disparities in health: sociological contributions. J. Health Soc. Behav. 51 (1_Suppl. l), S15–S27. 10.1177/0022146510383838 20943580 PMC3468327

[B60] Xia Y.-y. Ding Y.-b. Liu X.-q. Chen X. m. Cheng S. q. Li L. b. (2014/08/01/ 2014). Racial/ethnic disparities in human DNA methylation. Biochimica Biophysica Acta (BBA) - Rev. Cancer 1846 (1), 258–262. 10.1016/j.bbcan.2014.07.001 25016140

[B61] Yudell M. Roberts D. DeSalle R. Tishkoff S. (2016). Science and society. Taking race out of human genetics. Science 351 (6273), 564–565. 10.1126/science.aac4951 26912690

